# Stereoacuity and ocular-associated factors in children aged 3–7 years in Guangxi, Southwest China: a cross-sectional study

**DOI:** 10.3389/fmed.2025.1562041

**Published:** 2025-07-07

**Authors:** Xin Xiao, Huiyao Huang, Yan Luo, Wuqiang Luo, Lili Li, Enwei Lin, Min Kong, Qi Chen

**Affiliations:** ^1^Visual Science and Optometry Center of Guangxi, The People’s Hospital of Guangxi Zhuang Autonomous Region, Nanning, China; ^2^Guangxi Key Laboratory of Eye Health, The People’s Hospital of Guangxi Zhuang Autonomous Region, Nanning, China; ^3^Department of Scientific Research, The People’s Hospital of Guangxi Zhuang Autonomous Region, Nanning, China

**Keywords:** children, stereoacuity, visual acuity, anisometropia, astigmatism

## Abstract

**Objective:**

This study aimed to investigate the distribution of stereoacuity and its ocular-associated factors in children aged 3–7 years in Guangxi, Southwest China.

**Methods:**

This cross-sectional study recruited 4,090 children aged 3–7 years (mean: 5.12 ± 0.95 years) from 12 randomly selected kindergartens via cluster sampling in Nanning City, Guangxi, Southwest China. Comprehensive ocular assessments included visual acuity assessment, cover/uncover and alternating cover tests, anterior segment examination, fundus examination, the Titmus stereo test, and cycloplegic autorefraction. The univariate and multivariate logistic regression models were used to determine the factors associated with subnormal stereoacuity (>40 arcsec).

**Results:**

The prevalence rates of anisometropia, astigmatism, and strabismus were 18.24, 26.11, and 0.20%, respectively. The mean stereoacuity was 1.88 ± 0.34 log units (median: 60.25 arcsec), with the majority (65.18%) having subnormal stereoacuity. The mean log units of stereoacuity decreased with age (*F* = 144.7, *p* < 0.001). Compared to girls, boys had a significantly greater mean log unit stereoacuity (1.90 ± 0.35 vs. 1.87 ± 0.34, *t* = 2.589, *p* = 0.010). In the multivariate logistic regression, older age (odds ratio [OR]: 0.040–0.461 for years 4–7, 95% confidence interval [CI]: 0.018–0.613 for years 4–7, all *p* < 0.001) and girls (OR = 0.672, 95% CI: 0.584–0.772, *p* < 0.001) were protective factors, whereas interocular acuity difference [IAD] (OR = 6.906, 95% CI: 3.133–16.01, *p* < 0.001), mean LogMAR acuity (OR = 11.491, 95% CI: 6.065–22.153, *p* < 0.001), mean cylindrical error [CYLmean] (OR = 1.201, 95% CI: 1.055–1.365, *p* = 0.005), and anisometropia (OR = 1.452, 95% CI: 1.202–1.760, *p* < 0.001) were risk factors for subnormal stereoacuity.

**Conclusion:**

Ocular factors, including higher IAD, worse acuity, greater astigmatism, and greater anisometropia, were identified as risk factors for subnormal stereoacuity, highlighting the importance and urgency of early screening for stereoacuity and ocular risk factors in children aged 3–7 years in Guangxi.

## Introduction

Stereoacuity, the ability to perceive depth from differential retinal image positions in both eyes (1), is a fundamental binocular visual function (2, 3). It is critical for various aspects of daily life, including motor skill development (e.g., Purdue pegboard tasks, bead threading, water pouring, and surgical performance) (4) and academic performance (5, 6). It is particularly vital during childhood, as it influences walking (7, 8), quality of life (9, 10), and later-life skills such as driving and professional performance (11, 12). Normal stereoacuity relies on the proper functioning of the ocular, neural, and motor components, making it a valuable indicator of overall visual health (13, 14). In clinical settings, quantifying stereoacuity is crucial for managing strabismus and amblyopia and evaluating treatment outcomes in pediatric trials (15, 16).

Although controversy still exists over the exact age at which stereoscopic vision reaches maturity and stability (17, 18), it is currently widely believed that preschool and elementary school are critical periods for the development and maturity of stereoacuity (19, 20). Early identification of ocular risk factors during these stages is essential for healthcare providers to promote healthy stereoacuity development.

Interocular symmetry is crucial for stereoacuity and binocular vision (21). Previous studies in China have elucidated the stereoscopic development and ocular parameters of school-aged children (16, 19, 20). However, data on the ocular parameters that affect interocular symmetry and their relationships with stereoacuity are limited (21, 22). These parameters, including the interocular difference in spherical equivalent (SE), the interocular difference in cylindrical error, the interocular difference in spherical error, the interocular acuity difference (IAD), and strabismus, have not been extensively investigated, particularly in larger sample sizes or across broader age groups (23). In addition, our previous study revealed that high astigmatism is common among children aged 3–7 years in Guangxi, and magnitude-and orientation-dependent correlations between astigmatism and visual acuity have been confirmed (24). It is worth exploring whether children with ocular-associated factors (such as IAD, strabismus, and high astigmatism) have a greater prevalence of subnormal stereoacuity.

This cross-sectional, school-based study was conducted in Guangxi, Southwest China. It aimed to explore the distribution of stereoacuity and ocular parameters, such as anisometropia, astigmatism, IAD, and strabismus, in children, and to investigate the correlations between stereoacuity and ocular parameters. The research findings provide a basis for considering stereoscopic vision assessment as an indicator in children’s vision screening.

## Methods

This cross-sectional study was conducted in Nanning City, Guangxi, Southwest China, from 1 May to 30 October 2020. The sample size was calculated on the basis of a projected 18.5% prevalence of subnormal stereoacuity in 7-year-olds (16), aiming for a 95% confidence level and a 20% error margin. Using the formula (25): *n* = (Z^2^ * *ρ* * (1 − ρ))/B^2^, where ρ = 0.185, *B* = 0.185 * 0.20 = 0.037, and *Z* = 1.96, an initial sample of 2,115 children was required for a uniform age distribution across the 3-to 7-year range. Adjustments for a 20% non-response rate and a 25% increase due to random cluster sampling led to a revised minimum sample size of 3,304 participants.

**Figure 1:**
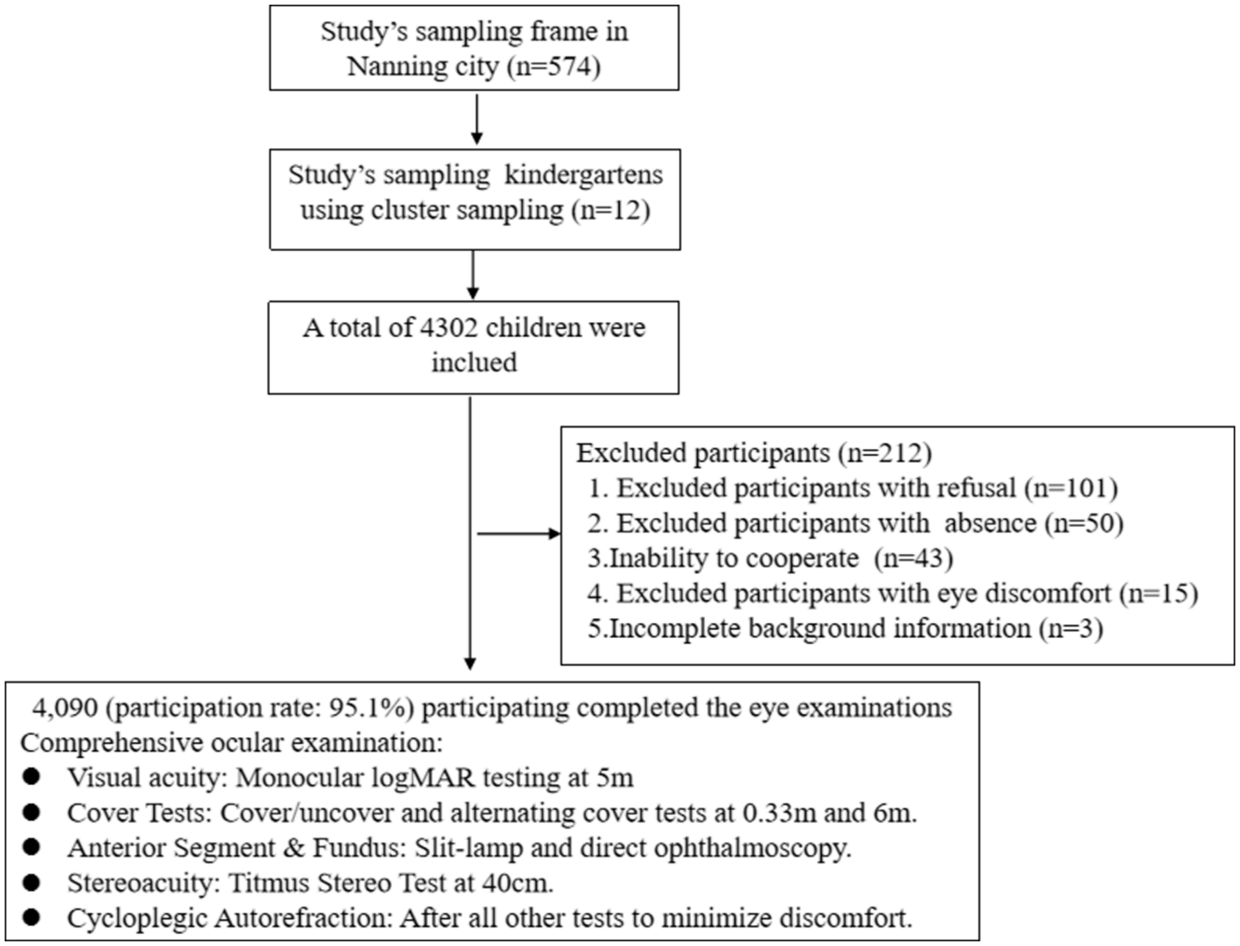
Flowchart of this study.

The study’s sampling frame encompassed 574 kindergartens in Nanning City, of which 12 were randomly selected through school-based cluster sampling. A total of 4,302 children were initially chosen, with 4,090 (participation rate: 95.1%) participating in the eye examinations. A total of 212 participants (4.9%) were excluded, primarily because of refusal (101), absence (50), inability to cooperate (43), eye discomfort (15), or incomplete background information (3). The study flowchart is presented in Figure 1.

### Ocular examinations

The children underwent a comprehensive ocular examination, which included visual acuity assessment, cover/uncover and alternating cover tests, anterior segment examination, fundus examination, the Titmus stereo test, and cycloplegic autorefraction (16, 19, 20).

Visual acuity was tested monocularly by nurses (HYH) via an E-letter standard logarithmic visual acuity chart (SJ-LED-01, Guangzhou Shihai Medical Corporation, Guangzhou, China) at 5 m. Visual acuity was expressed in decimal notation and analyzed via the logarithm of the minimum angle of resolution (logMAR) equivalents.

The cover/uncover and alternating cover tests at near (0.33 m) and distances (6 m), administered by experienced pediatric ophthalmologists (QC, YL, WQL, FX, and MK), were used to diagnose children with strabismus.

The anterior segment and ocular media were examined using skilled optometrists (EWL and XX) with a slit lamp microscope, whereas fundus examinations were performed via a direct ophthalmoscope (Beta 200; Heine Optotechnik GmbH & Co., Herrsching, Germany).

Stereoacuity was precisely evaluated using the Titmus stereo test (Stereo Optical Co., Inc., Chicago, IL, United States), following the manufacturer’s instructions (16, 19, 20). The Titmus test includes disparities ranging from 800 to 40 arcsec with unequal step sizes (800, 400, 200, 140, 80, 60, 50, and 40 arcsec). Through a pair of polarizing glasses, subjects viewed the stereogram at a distance of 40 cm and were asked to grasp the wings of the fly. The polarizing glasses were worn over the child’s prescription spectacles, if applicable. If the participant attempted to seize the wings of the fly, they were asked to point to the circle that appeared to “jump” out of the book. If an incorrect response was given, the previous target was reassessed. If an accurate response was repeatedly obtained for the preceding target, the target’s disparity was regarded as the stereoacuity value. The stereoacuity examinations were performed by a small group of trained optometrists (EWL, XX, and LLL) who were instructed to carry out the test under standardized conditions during the daytime. To facilitate clinical interpretation, stereoacuity scores in arcsec were initially transformed to logarithmic values (23).

An autorefractor (KR8900, Topcon, Tokyo, Japan), known for its precision and reliability (26), was used to perform refraction with cycloplegia. Cycloplegia was carried out using three drops of cyclopentolate hydrochloride 1% (Colircusí cycloplegic, Alcon Healthcare S. A., Barcelona) (27). Considering that cycloplegic refraction may cause temporary near vision blurriness and photophobia, autorefraction with cycloplegia was measured in the last step. Three consecutive measurements were taken, with the average value used for analysis.

### Definition

The spherical equivalent (SE) was calculated as the sum of the spherical refractive error and half of the cylindrical error (SE = sphere + 0.5 × cylinder), adhering to the International Myopia Institute (IMI) criteria (28). Myopia was classified by SE values of ≤ −0.50 diopters (D), whereas hyperopia was designated for SE values of ≥ +2.00 D. Astigmatism was defined as a cylindrical diopter of ≥ 1.00 D, and anisometropia was defined as a spherical equivalent difference or cylindrical error difference between the two eyes of at least 1.00 D. Mean logMAR acuity represented the average of binocular visual acuity in logMAR units. The interocular acuity difference (IAD) quantified the variation in visual acuity between eyes, expressed in logMAR. IAD was calculated as the absolute difference in best-corrected visual acuity (logMAR) between eyes: IAD = |logMAR_OD_-logMAROS|, where OD = right eye and OS = left eye. Interocular differences in the spherical refractive error (IDS), cylindrical error (IDC), and spherical equivalent (IDSE) were also assessed. SPHmean, CYLmean, and SEmean denote the means of the binocular spherical error, cylindrical error, and spherical equivalent, respectively, with interocular differences reported as absolute values. Strabismus was diagnosed if any tropia, phoria, or microtropia (esotropia, exotropia, or vertical) was observed at either a distance (6 m) or near (0.33 m). Subnormal stereoacuity was operationally defined as a stereoacuity score exceeding 40 arcseconds (16, 19).

### Quality control

Stringent quality control procedures were rigorously enforced throughout the study. Each participant received a thorough explanation of the procedures, and informed consent was obtained from participants before the assessments. The fieldwork was supervised by two ophthalmologists (WL and YL) to ensure strict adherence to the established protocols. A random 5% sample of the database was cross-checked for consistency by comparing original documents with electronic records. Data entry was independently performed by two individuals (HH and HL) using EpiData software 3.1 (The EpiData Association, Odense, Denmark), with discrepancies between the databases being resolved through rechecking. Standard operating procedures were developed and implemented for staff training to maintain uniformity and data accuracy.

### Statistical analysis

Data analysis was conducted via R programming (version 4.3.2). The normal distribution of the quantitative data was assessed using the Shapiro–Wilk test, and the data are presented as the means ± SDs for normally distributed data or medians (Q1 and Q3) for non-normally distributed data. Qualitative data were expressed as counts and percentages (N [%]). Independent *t*-tests were used to compare the differences in stereoacuity between the two groups, such as boys and girls. A one-way ANOVA was used to compare the differences in stereoacuity among different age groups. The chi-square test was used to compare the prevalence of subnormal stereoacuity among different groups.

We hypothesized that the log unit of stereoacuity may be associated with age, gender, and ocular measurement of children, with ocular parameters of interocular symmetry, such as, IDSE, IDC, IDS, IAD, and strabismus, and with parameters of refractive error, including SPHmean, CYLmean, SEmean, myopia (SE < −0.50D), hyperopia (SE ≥ +2.00D), anisometropia (IDSE ≥ 1.00D or IDC ≥ 1.00D), and astigmatism (cylindrical diopter≥1.00D).

Pearson’s or Spearman’s correlation analysis was used to assess the relationships between these parameters and log-transformed stereoacuity. To further explore the correlation between ocular parameters and stereoacuity, univariate and multivariate logistic regression models were used to calculate odds ratios (ORs) and their 95% CIs for factors associated with subnormal stereoacuity (>40 arcsec) (16, 19). All statistical tests were two-tailed, and *p*-values of < 0.05 were considered statistically significant.

## Results

### Characteristics of the subjects

A total of 4,090 children were enrolled, of which 2,169 (53.03%) were boys. Table 1 displays the characteristics of the subjects; the mean age was 5.12 ± 0.95 years (median: 5.12 years, range: 3.05–7.71 years), and the SE had a mean of 0.76 ± 1.04 diopters (median: 0.88 diopters, range: −10.50 ± 6.13 diopters). The mean cylindrical error was 0.42 ± 0.42 diopters (median: 0.31 diopters, range: 0.00–5.50 diopters), and the mean logMAR acuity was 0.17 ± 0.15 (median: 0.15, range: −0.10 to 2.00).

**Table 1:** Characteristics of the subjects.

Variables	*N* (%)	Mean ± SD
Age (year)		5.12 ± 0.95
3	544 (13.30)	
4	1,283 (31.37)	
5	1,468 (35.89)	
6	747 (18.26)	
7	48 (1.17)	
Gender		
Boys	2,169 (53.03)	
Girls	1,921 (46.97)	
Mean logMAR acuity		0.17 ± 0.14
IAD		0.06 ± 0.12
SPHmean (D)		1.06 ± 1.10
IDS (D)		0.53 ± 0.64
CYLmean (D)		0.09 ± 0.59
IDC (D)		0.28 ± 0.34
SEmean (D)		0.76 ± 1.04
IDSE (D)		0.52 ± 0.62
Refractive status		
Hyperopia	295 (7.21)	
Emmetropia	3,444 (84.21)	
Myopia	351 (8.58)	
Anisometropia		
Yes	746 (18.24)	
No	3,344 (81.76)	
Astigmatism		
Yes	1,068 (26.11)	
No	3,022 (73.89)	
Strabismus		
Yes	8 (0.20)	
No	4,082 (99.80)	

SD, standard deviation; Q_1_, first quartile; Q_3_, third quartile; logMAR, logarithm of the minimum angle of resolution; IAD, interocular acuity difference; SPHmean, mean of binocular spherical errors; IDS, the interocular difference in spherical refractive error; CYLmean, mean of binocular cylindrical errors; IDC, the interocular difference in cylindrical refractive error; SEmean, mean of binocular spherical equivalent; IDSE, the interocular difference in spherical equivalent.

The prevalence rates of myopia, hyperopia, and emmetropia were 8.58, 7.21, and 84.21%, respectively. The prevalence rates of anisometropia, astigmatism, and strabismus were 18.24, 26.11, and 0.20%, respectively.

### Distribution of stereoacuity

Table 2 shows the distribution of stereoacuity stratified by age, gender, and ocular parameters. The mean stereoacuity was 1.88 ± 0.34 log units (median: 60.25 arcsec, range: 40–3,552 arcsec), with the majority (65.18%) of children exhibiting subnormal stereoacuity. Notably, the mean log units of stereoacuity decreased with age (*F* = 144.7, *p* < 0.001), and the prevalence of subnormal stereoacuity decreased with increasing age (*χ*^2^ = 427.65, *p* < 0.001). Compared to girls, boys had significantly greater mean log units (1.90 ± 0.35 vs. 1.87 ± 0.34, *t* = 2.589, *p* = 0.010). The prevalence rate of subnormal stereoacuity in boys was also greater than that in girls (67.87% vs. 62.16%, *χ*^2^ = 14.386, *p* < 0.001).

**Table 2:** Distribution of stereoacuity stratified by age, gender, and ocular parameters.

Variables	Stereoacuity (arcsec)										Subnormal stereoacuity		Log unit of stereoacuity	
	All	40	50	60	80	100	140	200	400	≥800	No	Yes (>40 arcsec)	Mean ± SD	Median (Q_1_, Q_3_)
All	4,090	1,424	426	325	628	562	144	264	221	96	1,424 (34.82)	2,666 (65.18)	1.88 ± 0.34	1.78 (1.60, 2.00)
Age														
3 years	544	64	32	34	76	142	28	76	56	36	64 (11.76)	480 (88.24) *	2.11 ± 0.44	2.00 (1.90, 2.30)
4 years	1,283	288	126	107	225	245	50	97	105	40	288 (22.45)	995 (77.55)	1.96 ± 0.35	1.90 (1.90, 2.00)
5 years	1,468	621	165	122	246	133	42	70	52	17	621 (42.3)	847 (57.7)	1.82 ± 0.29	1.70 (1.60, 1.90)
6 years	747	414	98	59	81	41	22	21	8	3	414 (55.42)	333 (44.58)	1.74 ± 0.22	1.60 (1.60, 1.78)
7 years	48	37	5	3	0	1	2	0	0	0	37 (77.08)	11 (22.92)	1.65 ± 0.13	1.60 (1.60, 1.60)
Gender														
Boys	2,169	697	222	193	342	321	76	138	121	59	697 (32.13)	1,472 (67.87) *	1.90 ± 0.35	1.78 (1.60, 2.00)
Girls	1,921	727	204	132	286	241	68	126	100	37	727 (37.84)	1,194 (62.16)	1.87 ± 0.34	1.78 (1.60, 2.00)
Refractive status														
Hyperopia	295	104	35	34	43	24	18	15	19	3	104 (35.25)	191 (64.75) *	1.86 ± 0.30	1.78 (1.60, 2.00)
Emmetropia	3,444	1,225	356	263	512	474	113	230	195	76	1,225 (35.57)	2,219 (64.43)	1.88 ± 0.34	1.78 (1.60, 2.00)
Myopia	351	95	35	28	73	64	13	19	7	17	95 (27.07)	256 (72.93)	1.93 ± 0.42	1.90 (1.60, 2.00)
Anisometropia														
Yes	746	206	88	60	139	107	30	58	34	24	206 (27.61)	540 (72.39)*	1.92 ± 0.37	1.90 (1.60, 2.00)
No	3,344	1,218	338	265	489	455	114	206	187	72	1,218 (36.42)	2,126 (63.58)	1.87 ± 0.34	1.78 (1.60, 2.00)
Astigmatism														
Yes	1,068	318	117	84	183	157	42	76	65	26	318 (29.78)	750 (70.22) *	1.91 ± 0.34	1.90 (1.60, 2.00)
No	3,022	1,106	309	241	445	405	102	188	156	70	1,106 (36.6)	1,916 (63.4)	1.87 ± 0.34	1.78 (1.60, 2.00)
Strabismus														
Yes	8	2	2	0	2	1	0	0	0	1	2 (25.00)	6 (75.00)	1.91 ± 0.43	1.80 (1.67, 1.93)
No	4,082	1,422	424	325	626	561	144	264	221	95	1,422 (34.84)	2,660 (65.16)	1.88 ± 0.34	1.78 (1.60, 2.00)

*Difference between groups is statistically significant (*p* < 0.05).

There were statistically significant differences in subnormal stereoacuity prevalence among children with hyperopia, emmetropia, and myopia (64.75% vs. 64.43% vs. 72.93%, *χ*^2^ = 10.176, *p* = 0.006). Statistically significant differences were found in the prevalence of subnormal stereoacuity between children with anisometropia and those without anisometropia (72.39% vs. 63.58%, *χ*^2^ = 20.471, *p* < 0.001), as well as between children with astigmatism and those without astigmatism (70.22% vs. 63.40%, *χ*^2^ = 15.888, *p* < 0.001). No statistically significant differences were found in the prevalence of subnormal stereoacuity between children with strabismus and those without strabismus (75.00% vs. 65.16%, *χ*^2^ = 0.045, *p* = 0.832).

### Factors associated with stereoacuity via correlation analysis

**Figure 2:**
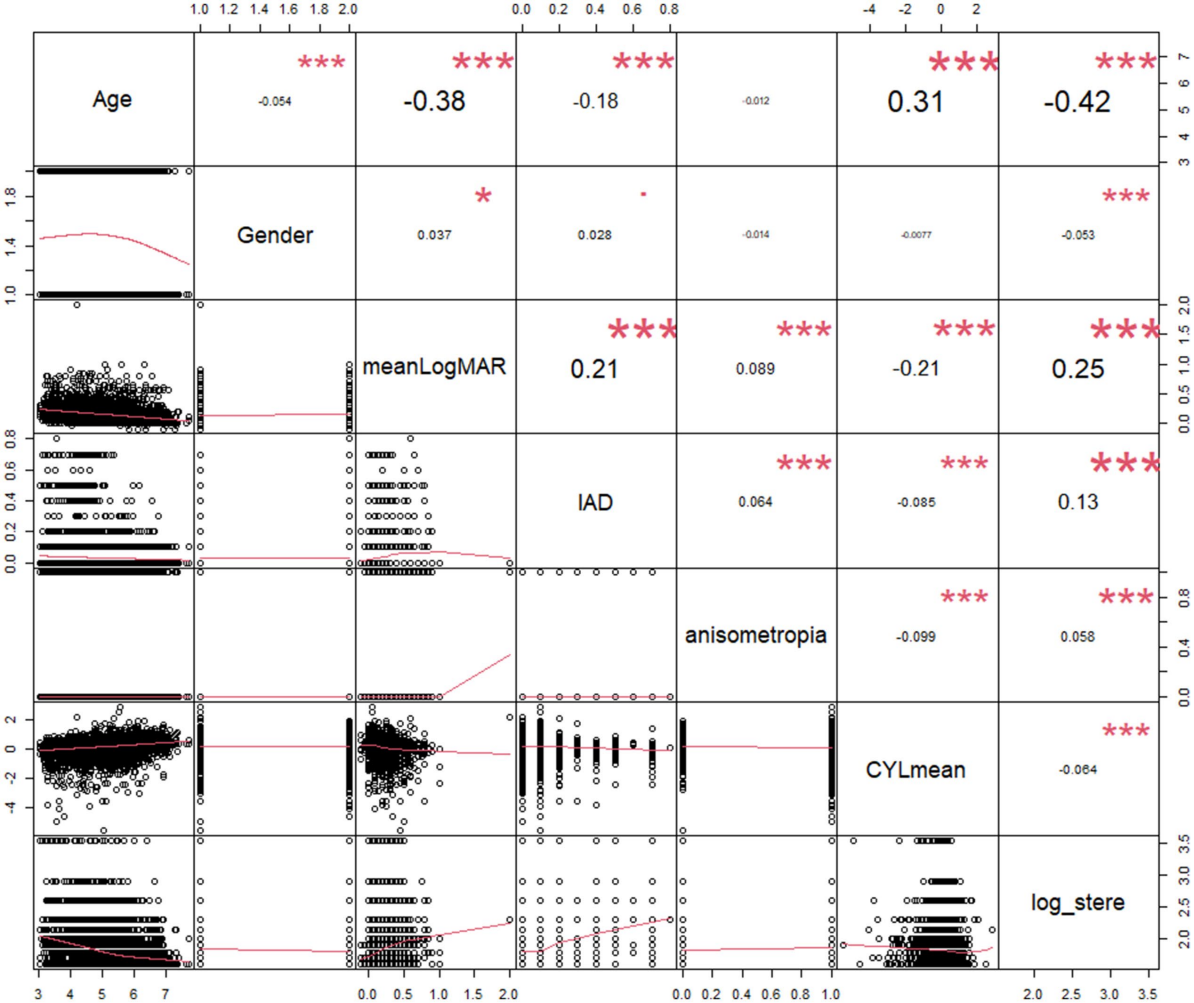
Scatterplot of the log value of stereoacuity with its correlates. IAD, interocular acuity difference; meanLogMAR, mean of binocular LogMAR; CYLmean, mean of binocular cylindrical errors; log_stere, log unit of stereoacuity.

According to the Pearson’s correlation analysis (Table 3; Figure 2), reduced stereoacuity was statistically correlated with younger age (*r* = −0.369, *p* < 0.001), boys (*r* = −0.053, *p* = 0.001), higher LogMAR acuity (worse visual acuity, *r* = 0.196, *p* < 0.001), increased IAD (*r* = 0.156, *p* < 0.001), IDS (*r* = 0.043, *p* = 0.006), IDC (*r* = 0.056, *p* < 0.001), IDSE (*r* = 0.048, *p* = 0.002), SEmean (*r* = 0.056, *p* < 0.001), decreased SPHmean (*r* = −0.043, *p* = 0.006), and decreased CYLmean (*r* = −0.051, *p* = 0.001). Spearman’s correlation analysis revealed that there was a significant correlation between reduced stereoacuity and refractive status (*ρ* = 0.039, *p* = 0.012), anisometropia (*ρ* = 0.058, *p* < 0.001), and astigmatism (*ρ* = 0.059, *p* < 0.001), whereas there was no significant association between stereoacuity and strabismus (*ρ* = 0.002, *p* = 0.897).

**Table 3:** Correlation analysis between stereoacuity (log unit) and ocular parameters.

Variables	Correlation coefficient (*r*/ρ)	*p* value
Age	−0.369	<0.001
Gender	−0.053	0.001
Mean logMAR acuity	0.191	<0.001
IAD	0.226	<0.001
SPHmean	−0.043	0.006
IDS	0.043	0.006
CYLmean	−0.051	0.001
IDC	0.056	<0.001
SEmean	0.056	<0.001
IDSE	0.048	0.002
Refractive status	0.039*	0.012
Anisometropia	0.058*	<0.001
Astigmatism	0.059*	<0.001
Strabismus	0.002*	0.897

IAD, interocular acuity difference; SPHmean, mean of binocular spherical errors; IDS, interocular difference in spherical refractive error; CYLmean, mean of binocular cylindrical errors; IDC, interocular difference in cylindrical refractive error; SEmean, mean of binocular spherical equivalent; IDSE, interocular difference in spherical equivalent.

### Factors associated with subnormal stereoacuity via logistic regression

In the logistic regression analysis, we incorporated age, gender, mean LogMAR acuity, IAD, IDS, IDC, IDSE, SPHmean, SEmean, CYLmean, refractive status, anisometropia, astigmatism, and strabismus as predictors of subnormal stereoacuity, and almost all the predictors, with the exception of strabismus, were statistically significantly associated with subnormal stereoacuity in the univariate model (all *p* < 0.05, Table 4). We further developed a multivariate logistic regression model with these significant predictors of univariate analysis and used a stepwise approach based on the Akaike information criterion (AIC) for selecting variables. We identified the final model with an AIC of 4711.5 and revealed that older age (OR: 0.040 ~ 0.461 for years 4–7, 95% CI: 0.018–0.613 for years 4–7, all *p* < 0.001) and girls (OR = 0.672, 95% CI: 0.584–0.772, *p* < 0.001) were protective factors against subnormal stereoacuity, whereas IAD (OR = 6.906, 95% CI: 3.133–16.01, *p* < 0.001), mean LogMAR acuity (OR = 11.491, 95% CI: 6.065–22.153, *p* < 0.001), CYLmean (OR = 1.201, 95% CI: 1.055–1.365, *p* = 0.005), and anisometropia (OR = 1.452, 95% CI: 1.202–1.760, *p* < 0.001) were positively associated with an increased likelihood of subnormal stereoacuity (Table 4).

**Table 4:** Univariate and multivariate logistic regression analyses for associations between ocular parameters and stereoacuity (log unit).

Characteristics	*N*	Subnormal stereopsis* n (%)	Univariate analysis			Multivariate analysis		
			OR	95%CI of OR	*P* value	OR	95%CI of OR	*P* value
Age								
3 years	544	480 (88.24)	Ref.			Ref.		
4 years	1,283	995 (77.55)	0.461	0.342–0.613	<0.001	0.528	0.389–0.706	<0.001
5 years	1,468	847 (57.7)	0.182	0.136–0.239	<0.001	0.224	0.166–0.297	<0.001
6 years	747	333 (44.58)	0.107	0.079–0.144	<0.001	0.139	0.101–0.189	<0.001
7 years	48	11 (22.92)	0.040	0.018–0.079	<0.001	0.049	0.022–0.100	<0.001
Gender								
Boys	2,169	1,472 (67.87)	Ref.			Ref.		
Girls	1,921	1,194 (62.16)	0.778	0.684–0.885	<0.001	0.672	0.584–0.772	<0.001
Mean logMAR acuity			63.5	34.20–120.00	<0.001	11.491	6.065–22.153	<0.001
IAD			34.1	15.7–77.9	<0.001	6.906	3.133–16.010	<0.001
SPHmean (D)			0.928	0.874–0.985	0.015			
IDS (D)			1.21	1.09–1.35	<0.001			
CYLmean (D)			0.751	0.667–0.884	<0.001	1.201	1.055–1.365	0.005
IDC (D)			1.58	1.28–1.96	<0.001			
SEmean (D)			0.887	0.831–0.946	<0.001			
IDSE (D)			1.21	1.08–1.35	0.001			
Refractive status								
Hyperopia	295	191 (64.75)	Ref.					
Emmetropia	3,444	2,219 (64.43)	0.986	0.767–1.26	0.091			
Myopia	351	256 (72.93)	1.47	1.05–2.05	0.025			
Anisometropia								
Yes	746	540 (72.39)	1.50	1.26–1.79	<0.001	1.452	1.202–1.760	<0.001
No	3,344	2,126 (63.58)	Ref.			Ref.		
Astigmatism								
Yes	1,068	750 (70.22)	1.36	1.17–1.58	<0.001			
No	3,022	1,916 (63.4)	Ref.					
Strabismus								
Yes	8	6 (75.00)	1.60	0.369–11.0	0.563			
No	4,082	2,660 (65.16)	Ref.					

^*^Subnormal stereoacuity was defined as stereoacuity worse than 40 arcsec (stereoacuity value >40 arcsec).

OR, odds ratio; CI, confidence interval; IAD, interocular acuity difference; SPHmean, mean of binocular spherical errors; IDS, the interocular difference in spherical refractive error; CYLmean, mean of binocular cylindrical errors; IDC, the interocular difference in cylindrical refractive error; SEmean, mean of binocular spherical equivalent; and IDSE, the interocular difference in spherical equivalent.

## Discussion

This large-scale, school-based cross-sectional investigation examined stereoacuity and its ocular correlates in children aged 3–7 years in Nanning City, Guangxi, Southwest China, a region with diverse ethnic minorities (29). The mean stereoacuity was 1.88 ± 0.34 log units (median: 60.25 arcsec), with 65.18% having subnormal (>40 arcsec) stereoacuity. The prevalence rates of anisometropia, astigmatism, and strabismus were 18.24, 26.11, and 0.20%, respectively. After adjusting for covariates, IAD (OR = 6.906, *p* < 0.001), mean LogMAR acuity (OR = 11.491, *p* < 0.001), CYLmean (OR = 1.201, *p* = 0.005), and anisometropia (OR = 1.452, *p* < 0.001) were risk factors for subnormal stereoacuity. Our findings contribute new insights into the distribution of stereoacuity and its associations with ocular parameters in children through the Titmus stereo test.

The test was completed by 95.1% of participants. The mean log unit of stereoacuity decreased with age (2.11 ± 0.44 log arcsec for 3-year-olds, decreasing to 1.65 ± 0.13 log arcsec for 7-year-olds, *F* = 144.7, *p* < 0.001), suggesting improved stereoacuity scores as children aged (1, 6), which is consistent with prior research (13, 19, 20). However, controversy still remains regarding the exact age at which stereoscopic vision reaches maturity and stability. Oduntan et al.’s study using the Randot Stereo test suggested a later maturation period for boys aged 6–12 years (30). Birch et al.’s research with the Randot Preschool Stereoacuity Test showed ongoing improvement until 10 years (15, 31). There is a contradiction between different studies on the precise age of stable and mature stereoacuity development, which may be explained by ethnic differences in study subjects (15, 21, 22, 31, 32) and differences in stereoscopic assessment methods (e.g., the Titmus, Randot, or Lang stereo test tools) (22, 32, 33). Our study’s correlation analysis and multivariate analysis results supported that age was correlated with stereoacuity, which emphasizes the importance of stereoacuity screening for children aged 3–7 years (20, 34). Further research is needed to clarify the health economic benefits of stereoacuity screening in this age group.

Our study revealed statistically significant gender differences, with boys exhibiting greater mean log units of stereoacuity than girls did (1.90 ± 0.35 vs. 1.87 ± 0.34 log arcsec, *t* = 2.589, *p* = 0.010). Gender was a significant predictor in the multivariate model, negatively correlated with stereoacuity (*r* = −0.053, *p* = 0.001), and associated with increased odds of subnormal stereoacuity in boys (OR = 0.672, 95% CI: 0.584–0.772, *p* < 0.001). This finding aligns with Potluri et al.’s research using the Titmus stereoacuity test in a sample of 2,376 children aged 7–14 years and reported slightly better stereoacuity in girls (31). In contrast, in their study of 942 children aged 4–5 years, Han et al., who used the same test, did not detect a significant gender association (19). Our results suggest that girls’ faster visual development during early childhood, as suggested by previous research (35), may contribute to this gender disparity.

Our findings are consistent with those of previous studies that have reported an association between lower visual acuity and weaker stereoacuity in different age groups (19, 20, 32, 36). Schmid et al. (36) and Sandfeld et al. (32) reported a connection between poor stereoscopic performance and reduced visual acuity in children, supporting our observations. A study of preschool children (19) revealed a correlation between improved visual acuity and higher stereoacuity scores. Furthermore, a study by Guo et al. (20) revealed a statistically significant association between a greater intereye difference in best-corrected visual acuity (logMAR) and reduced stereoacuity in a cohort aged 4–18 years, thereby corroborating our results.

This study revealed a high prevalence of astigmatism in children in Guangxi (26.11%), which confirmed our previous findings (24). Our multivariate logistic regression analysis revealed that, after adjusting for the mean logMAR acuity and IAD, an increase in the binocular cylinder error (CYLmean) was a significant risk factor for subnormal stereoacuity (OR = 1.201, 95% CI: 1.055–1.365, *p* = 0.005), indicating that astigmatism is an independent risk factor for stereoacuity, which is consistent with the findings of previous studies (23, 26, 37, 38). The association of astigmatism is believed to be due to factors such as optical blur, foveal suppression, or selective changes in the nerves within the VI cortex (39).

In addition, anisometropia was significantly correlated with stereoacuity (*r* = 0.058, *p* < 0.001), and the risk of subnormal stereoacuity among children with anisometropia was 1.452 times greater than that among those without anisometropia (OR = 1.452, 95% CI: 1.202–1.760, *p* < 0.001), highlighting the substantial effect of interocular refractive asymmetry on stereoacuity. The studies by Ying et al. (38) reported that a greater degree of anisometropia was associated with worse stereoacuity in a dose–response manner in children, supporting our observations. The mechanism by which anisometropia impairs children’s stereoacuity is primarily through interrupting normal binocular fusion (26, 40, 41).

Similarly, numerous studies have revealed that strabismus is another key factor affecting the development of stereoacuity, mainly by affecting interocular optical asymmetry to harm stereoacuity (23, 32, 42–44). However, our study used Spearman’s rank analysis and did not find a significant association between strabismus and subnormal stereoacuity. This deviation from the established association may be attributed to the extremely low prevalence of strabismus (0.20%, with only eight children suffering from strabismus) in Nanning children. Our strabismus prevalence (0.20%) may underrepresent intermittent exotropia due to single-time point screening. Future studies should incorporate repeated cover tests or home-based assessments to capture transient deviations.

The robustness of our study is substantiated by its large sample size, high response rate, inclusion of age-specific participants, comprehensive assessments, and adherence to standardized procedures supervised by ophthalmologists, optometrists, and medical professionals. Despite these strengths, several limitations should be acknowledged. First, the reliance of the Titmus stereo test on monocular cues might enable several children to provide correct responses through these cues (45). In addition, the clinical value of stereoacuity screening lies in the fact that it may indicate potential visual system issues, including refractive errors, strabismus, and anisometropia. Stereoacuity testing can reveal subtle deficits in depth perception that might not otherwise be detected. In some cases, even a mild impairment of stereoacuity may indicate an increased risk of other visual or developmental problems later in life. For example, children with poor stereoacuity may be more likely to experience academic or developmental delays due to difficulties with spatial awareness and hand–eye coordination (46).

However, a previous study indicated that the influence of monocular cues on stereoacuity is minimal and thus unlikely to significantly affect the multivariate regression results (26). The accuracy of stereoacuity measurements via the Titmus test depends on children’s focus and cognitive abilities (1), which, given their developing nature at preschool age (47, 48), may impact the observed correlations between stereoacuity and accuracy. Second, as our study is cross-sectional, future research with a longitudinal cohort design and a substantial sample size will be necessary to further validate our findings and enhance their generalizability. Third, the 12 kindergartens in this study were all located in the urban area of Nanning, and the sample lacked socioeconomic composition (no questionnaire survey), which limits the external applicability of the research results.

In conclusion, our study demonstrated that the mean stereoacuity was 1.88 ± 0.34 log units (median: 60.25 arcsec), with 65.18% of children exhibiting subnormal stereoacuity (>40 arcsec). The prevalence rates of anisometropia, astigmatism, and strabismus were 18.24, 26.11, and 0.20%, respectively. A significant correlation was observed between reduced stereoacuity and ocular factors, including age, gender, mean LogMAR acuity, IAD, IDS, IDC, IDSE, SPHmean, SEmean, CYLmean, refractive status, anisometropia, and astigmatism, as predictors of subnormal stereoacuity, with the exception of strabismus. Age and gender were identified as protective factors, while increased interocular acuity difference (IAD), mean logMAR acuity, mean binocular cylindrical error (CYLmean), and anisometropia were risk factors for subnormal stereoacuity after adjusting for covariates. Therefore, these factors should be recognized as important predictors of subnormal stereopsis. The clinical value of stereoacuity screening lies in its potential to suggest certain underlying visual system disorders in school screening, especially in cases where an ophthalmologist is not available. To reinforce these findings, future research should focus on longitudinal, multicenter studies with large sample sizes to enhance generalizability.

## Data Availability

The raw data supporting the conclusions of this article will be made available by the authors, without undue reservation.
